# Determinants of emotional exhaustion among nursing workforce in urban Ghana: a cross-sectional study

**DOI:** 10.1186/s12912-020-00512-z

**Published:** 2020-12-07

**Authors:** Collins Atta Poku, Ernestina Donkor, Florence Naab

**Affiliations:** 1 Department of Nursing, Kwame Nkrumah University of Science and Technology, Kumasi, Ghana; 2 School of Nursing and Midwifery, University of Health and Allied Sciences, Ho, Ghana; 3 School of Nursing and Midwifery, University of Ghana, Legon-Accra, Ghana

**Keywords:** Determinants, Emotional exhaustion, Nursing workforce, Urban Ghana

## Abstract

**Background:**

The subject of emotional exhaustion organisations has become important because of the emerging trends in employment and its associated challenges. Unhealthy practice environment is a major threat in the incidence of emotional exhaustion among nurses; and any organisational culture that do not support its personnel has huge burnout costs. The study aimed at assessing rate of emotional exhaustion; determining factors that accounts for it and also ascertaining the coping strategies used by nurses to overcome it in the Ghanaian health care setting.

**Methods:**

A cross-sectional study with a proportionate stratified sampling was used to draw a sample from five health facilities. A standardized questionnaire of Professional Practice Environment Scale of Nursing Work Index, Maslach Burnout Inventory and Coping Scale were used to assess variables under study. The STROBE guidelines were followed in reporting this study.

**Results:**

Out of the 232 registered nurses studied, 91.1% of them reported experiencing moderate to high rate of emotional exhaustion. The practice environment of the nurses explained 39.6% of the variance in emotional exhaustion. Emotion-focused and problem-focused approaches were identified to be used by registered nurses to cope with emotional exhaustion.

**Conclusion:**

When appropriate and effective intervention are employed, emotional exhaustion will be reduce and this will enrich the effectiveness of quality care delivery to patients.

## Background

The subject of burnout in a form of emotional exhaustion has become imperative in health organizations because of the emerging trends in employment and its related problems. It is been ascertained that unhealthy practice environment such as increased workloads, absurd nurse-patient ratios, and scarce human and material resources is a major threat in the well-being of professionals especially in the incidence of emotional exhaustion among nursing workforce [1–4]; and any organisational culture that does not support its personnel has burnout costs [4]. Emotional exhaustion, which is one of the pillars of ‘Burnout Syndrome’ is defined as syndrome characterized by losing enthusiasm for work [5–8] and it is conceptualised as a response to a discrepancy between job-related strains and resources that is presented through feelings of emotional fatigue [9]. Emotional exhaustion also presents as a chronic manifestation of somatic and emotional depletion that results from extreme workload and/or personal strains and incessant tension from job [10]. It is understood to develops from defects in the structural and psychological make-ups in an organisation [11, 12]; and the study of this phenomenon in other jurisdictions among nurses have produced distinctive outcomes that need a critical look.

Among health workers, the nursing workforce is mostly found to report constantly complex intensity of emotional exhaustion. The frequency ranges between 37 and 89% among nurses in Sub-Saharan Africa (SSA). It is, however, common among Intensive Care Units (ICUs) nurses and those who provide reproductive health services. The resultant consequences are low staff productivity and dissatisfaction of care provided by health caregivers [13, 14]. Within the context of a hospital setting, nurses in various units are duty bound to provide care to patients in settings associated with complex care and impending death. They also face death and grief situations on daily basis, and are at risk of becoming more susceptible to psychological repercussions and stress, frequently occasioning in emotional exhaustion [15, 16]. Emotional exhaustion of nurses has been identified to increased work-related infections, increased medication error, high incidence of patients’ fall, poor nurse-physician relations, low personal accomplishment of the nurse, job dissatisfaction and increased turnover intention [17, 18]. Burnout at workplace also gives rise to chronic adverse emotions such as anger, anxiety or depression, mental exhaustion, apprehension, low enthusiasm, and absenteeism which certainly endangers not only the nurses own health, but also their patients [19, 20].

In low-middle-income countries (LMICs) where nurses undertake their task in unfavourable practice environment (uncomfortable postures, extreme noise intensities, and congested work area), there is moderate to higher dimensions of burnout in the form of emotional exhaustion [21]. This obviously make the responsibility of giving care to patients extra demanding. The interactions between professional’s work environment and emotional exhaustion cannot be underestimated; as workplace which boast of quality leadership and nurses’ involvement in decision-making presents with low levels of exhaustion [22, 23]. Greater autonomy for nurses in respect of adequate staffing and resources and positive nurse-physician relation has also been found to improve this phenomenon; and thus reflects on nurses’ job satisfaction and enhanced quality of care to clients [24–26].

With World Health Organization (WHO) report on nursing workforce stating that more nurses are required in attaining satisfactory nurse-patient ratio especially in LMICs [27]; measures are needed to reduce emotional exhaustion among nurses. Potential widening of the disproportion of nurse-patient ratio places huge workload that build continuous psychosomatic stress on nurses in a form of fatigue and emotional exhaustion. In extreme cases, emotional exhaustion is reported to results in work-family conflict for the nurse [28, 29].

In SSA, organisational factors (demands from job, control over work, social support, and interpersonal relationships, and change over the role of staff) considerably impact on staff’s emotional exhaustion. Most nurses have increased chances of exhaustion relative to other health care providers [30, 31]. As reported in Switzerland 1:59, Canada 1:106 and United Kingdom 1:118; there is better efficiency in relation to nurse-patient ratio in high-resource countries compared to reported lower nurse-patient ratio in countries in SSA, with perhaps the exception of South Africa (1:192) and few other countries with encouraging statistics. Evidently, Sudan (1:833), Gambia (1:1111), Rwanda (1:1250) and Mali (1:1667) reported a very low nurse-patient ratio with Senegal (0.3) and Mozambique (0.4) even more frightening. While there has been significant improvement in the nurse-patient ratios in Ghana from 1:1251 in 2012 to 1:542 in 2016, and currently 4.2:1000, much is still needed to increase the nursing workforce in Ghana. With this unsafe nurse-to-patient ratio in most health care facilities in Ghana, the expected result is increased levels of stress among health staff [32]. Overall, the key implication is that most countries in SSA will not be able to attain the WHO’s recommendation of nurse-patient ratio of 1:300; and thus further increase episodes of emotional exhaustion and decreased quality of care to patients in this part of the world [33–35].

Complains of inadequate recognition from clients and their relations and the larger society as a whole usually serve as the main cause of nurses’ emotional exhaustion. Additionally, some health care practitioners also experience emotional exhaustion in a form of despair when their patients relapse or their condition deteriorate despite the amount of time and quality of care provided to them [36]. Importantly, it has been an established fact that challenges of role ambiguity of nurses who are promoted without adequate training on their new roles usually cause an upsurge in job burdens. Such situations increase the amount of work by nurses; and inadequate human and material resources to perform these roles result in emotional exhaustion [37]. Emotional fatigue compromises nursing care; thus delay in the recuperating time of patients and avoidable deaths; and also affect nurses’ loyalty to an organisation thereby increasing turnover intentions [38].

Emotional exhaustion among nurses in Ghana has not been given considerable attention, as most studies are directed towards other work-related risks with emphasis placed primarily on healthcare professionals in general. Besides, in circumstances where it has been studied, it is generally restricted to a few categories of nurses; therefore, the trends among the broad segment of nursing workforce cannot be evaluated. Additionally, in order to ameliorate the hazards caused by emotional exhaustion, and its compounding effects on job satisfaction, quality nursing care and turnover intention; it would be appropriate for nurse researchers to devote more attention to undertaking studies that will holistically assess this concept. The study therefore, aimed at assessing rate of emotional exhaustion, determining the factors that accounts for it and also ascertaining the coping strategies used to overcome it among Ghanaian nurses.

## Methods

### Study settings

The study was conducted at Sunyani Municipality, which is situated in the middle part of the country. It is the capital of one of the 16 regions in Ghana (Bono Region). Sunyani is an urban district with population of 123,224. The municipality occupies a land area of 1289 km^2^, and has hospitals [6], clinics [12], Community-based Health Planning and Services (CHPs) compound [7], maternity homes [3] and health centers [3]. These facilities provide health services to the population [39]. The facilities in Sunyani Municipality serve as the treatment for inhabitants of Sunyani and adjacent districts and referral center for other facilities in the surrounding regions of Ahafo, Bono East, and Northern part of Ghana. The municipal’s nursing workforce stands at 480; and they comprises of all categories of nurses: general nurses, midwives, psychiatric nurses, and other specialist nurses [40].

### Study design and sample size

Quantitative descriptive cross-sectional design was employed to obtain data through standardized questionnaires. Registered nurses from five [5] healthcare facilities in the Sunyani Municipal, Ghana were chosen for the conduct of the study. The population comprised Registered Nurses in the selected facilities, with inclusion criteria being nurses who have worked for at least one-year after qualification from Nursing and Midwifery Council of Ghana and were willing to participate in the study. Registered nurses were excluded if they were on annual leave at the time of the study. Miller and Brewer’s mathematical formula (*n* = N/1 + Ne^2^) for estimating sample size was used; where n is the sample size, N is the total population, and e is the margin of error. In effect, the standard deviation was set at 95% confidence level with a margin of error of 0.05. The sample size was increased and rounded up with 10% of the calculated minimum sample size to take care of non-responses, inappropriately filled and/or missing questionnaires. A total of 250 questionnaires were distributed for the study, however, 232 were completely filled and returned; representing 92.8% response rate.

### Sampling technique

A proportionate stratified sampling technique was used to obtain the required number of respondents from each of the selected facilities based on the inclusion criteria. Thus, respondents from each stratum were made to randomly pick ballot labelled ‘YES’ to be selected as part of the study and ‘NO’ to be excluded from the study. This was done to avoid respondent selection bias.

### Data collection

Self-administered questionnaire was done and the process lasted for 3 weeks (4th April, 2017 – 21st April, 2017). Respondents used approximately 30 min to fill the questionnaire. Standardized tools were adapted for the study of the various variables. The Professional Practice Environment was measured using the “*Practice Environment Scale of Nursing Work Index (PES-NWI)”* developed by Lake (2002) [41]. The tool is made up of 5 sub-scales (nurse manager ability, leadership and support, collegial nurse-physician relations, staffing and resource adequacy, nurse participation in hospital affairs and nursing foundations for quality care) with sum of 32 items on a four Likert scale 1–4 (1 = strongly disagree; 4 = strongly agree) was used to measure PPE. The total score for Professional Practice Environment is the sum of all 32 items with a range of 32 to 128. Scores between 1 and 32 indicated poor practice environment; a score between 33 and 85 showed moderate practice environment while scores above 85 showed good practice environment. Most studies have used this scale and it has proven satisfactory reliability [42, 43]. The emotional exhaustion dimension of the *“The Maslach Burnout Inventory (MBI)”* Scale developed by Maslach, Jackson, and Leiter (1981) was used to measure emotional exhaustion of nurses. It is made up of 9 items and measured on a seven Likert scale (0 = Never; 6 = Everyday). Items summed and averaged to provide emotional exhaustion score. A score of 0 to 16, 17 to 26, and 27 and above indicated low, moderate and high emotional exhaustion respectively. Earlier studies which used the MBI demonstrated acceptable Cronbach’s alpha [44]. Coping strategies used by nurses were measured using the *“Coping Scale”* [45]. It is measured on a four Likert scale (4 = Mostly true about me; 1 = Not true about me); and made up of 13 items. Higher scores indicate higher level of coping. The scale has a reliability of 0.69 [46].

In order to validate the tool, a pre-test of the standardised questionnaire was conducted on 20 nurses from SDA Hospital-Sunyani who met the inclusion criteria, and it yielded reliability of 0.86. This exercise was done to ascertain the appropriateness of the questionnaire, whether it is difficult to understand or whether questionnaire contained culturally insensitive questions. To ensure reliability of the tool, the various part of the questionnaire was adapted except socio-demographic data. The Cronbach’s alpha for the scale was estimated as 0.86 after the test with component subscales as follows: PES-NWI - 0.88, MBI - 0.90 and Coping Scale of 0.74, all considered standard [47].

### Ethical approval and consent to participate

An ethical clearance was sought from the Noguchi Memorial Institute for Medical Research Institutional Review Board –IRB with number CPN 045/16–17 while respondents also gave verbal consent prior to data collection as approved by the IRB. Verbal consent was used as most participants were skeptical about undertaking written consent. The benefits and possible risks were also explained to respondents. Additionally, respondents’ anonymity and confidentiality were assured by indicating that they were not required to write their name on the questionnaire and by assuring them that their responses will not in any way be linked to them. Respondents were told that participation was voluntary and that they were free not to respond to questions they did not want. They were also informed that the completion of the questionnaire indicated explicit consent to use the data for research purposes.

### Data analysis

Statistical Package for Social Sciences (SPSS), version 23.0 was used for the data analysis. Descriptive statistics (means, frequencies and standard deviations) were used to summarise the variable of interest. Pearson correlation analysis was conducted to determine the relationship between professional practice environment and emotional exhaustion while the two models of multiple regression analysis was used to determine the predictors of emotional exhaustion of nurses. Also, mean and standard deviation were used to analyse the coping strategies used by nurses. Data analysis was conducted at a significance level of 0.05 and power of 95%.

## Results

### Socio-demographic characteristics

The socio-demographic characteristics of the 232 respondents are presented in Table 1 below. The study showed majority of registered nurses (62.1%) were between the ages of 26 and 35 years, with the mean age of 29.13 (SD = 5.061). Approximately 71% (*n* = 164) were females, about half of them (50.4%) were married while 191 (82.3%) had diploma in nursing education. The average working years for the respondents was 3.71 with most of them (83.6%, *n* = 194) having worked for a period of 1 to 6 years. In addition, 34% (*n* = 79) of the nurses worked at the Medical/Surgical Units, and more than half (56.6%) of the respondents were staff nurses. Most of the nurses (47.8%) attend 8-h shift duty. The mean score for emotional exhaustion was high (mean = 31.24, SD = 13.29); correspondingly most respondents (91.1%, *n* = 209) experienced moderate to high forms of emotional exhaustion at work.

**Table 1:** Socio-demographic characteristics of respondents

Variable (***N*** = 232)	Frequency (n)	Percent (%)
Age of respondent		
18–25	59	25.4
**26–35**	**144**	**62.1**
36–45	25	10.8
45–59	4	1.7
Gender		
Male	68	29.3
**Female**	**164**	**70.7**
Marital status		
Single	113	48.7
**Married**	**117**	**50.4**
Separated	2	0.9
Educational Qualification		
**Diploma**	**191**	**82.3**
Bachelor	33	14.2
Masters	8	3.4
Years in Nursing		
**1–3 year**	**106**	**45.7**
v4–6 years	88	37.9
7-10 years	24	10.3
More than 10 years	14	6.0
Years in the hospital		
**1–3 year**	**104**	**44.8**
4–6 years	98	42.2
7-10 years	22	9.6
More than 10 years	8	3.4
Area of work in hospital		
Critical care/Emergency	38	20.7
**Medical unit**	**46**	**19.8**
Surgical unit	33	14.2
Obstetrics	31	13.4
Pediatric	28	12.1
Theatre/ICU	17	7.3
Orthopedics	6	2.6
Mental Health/Psychiatry	14	6.0
Others	9	3.9
Current rank of respondent		
**Staff Nurse/Midwives**	**131**	**56.6**
Head nurse	59	25.4
Charge nurse	26	11.2
Supervisor	13	5.6
Directors	1	0.4
Others	2	0.9
Type of shift system by respondent		
**8 h**	**111**	**47.8**
12 h	15	6.5
Both 8 and 12 h	106	45.7
Emotional Exhaustion of Nurses		
Low	23	9.9
**Moderate**	**125**	**53.9**
High	84	36.2
Emotional Exhaustion of Nurses	**Mean** 31.24	**SD** 13.29

Source: Field Data (2017)

Table 2 above presents the correlation between selected demographic information, facets of practice environment and emotional exhaustion of registered nurses. The highest qualification of the nurse and all the facets of professional practice environment had significant negative correlations (*p < 0.05*) with emotional exhaustion as follow: highest qualification of the nurse (*r* = −.185), nursing foundation for quality of care (*r* = −.259), staffing and resource adequacy (*r* = −.517), nurse manager ability, leadership and support (*r* = −.164), collegial nurse-physician relation (*r* = −.243) and nurse participation in hospital affair (*r* = −.538).

**Table 2:** Correlation between selected variables and emotional exhaustion

Variables	EE	A	H	YN	YH	C	NMLS	NPR	NPHA	SRA	NFQC	PES
Emotional Exhaustion (EE)	1	.010	−.185^**^	−.003	.002	.066	−.164^*^	−.243^**^	−.538	−.517^**^	−.259^**^	−.338^**^
Age of nurse (A)	.010	1	.652^**^	.968^**^	.935^**^	.486^**^	.034	.027	.060	.033	−.051	.008
Highest qualification (H)	−.185^*^	.652^**^	1	.660^**^	.614^**^	.404^**^	.118	.232^**^	.104	.242^**^	.165^*^	.237^**^
Years in Nursing (YN)	−.003	.968^**^	.660^**^	1	.954^**^	.479^**^	.040	.031	.044	.027	−.060	.010
Years in hospital (YH)	.002	.935^**^	.614^**^	.954^**^	1	.484^**^	.069	.042	.051	.039	−.062	.018
Current rank of nurse (C)	.066	.486^**^	.404^**^	.479^**^	.484^**^	1	−.072	.055	.026	.029	−.068	.010
Nurse Manager Ability Leadership & Support (NMALS)	−.164^*^	.034	.118	.040	.069	−.072	1	.367^**^	.075	.359^**^	.280^**^	.382^**^
Nurse-Physician Relation (NPR)	−.243^**^	.027	.232^**^	.031	.042	.055	.367^**^	1	.409^**^	.892^**^	.749^**^	.936^**^
Nurses’ Participation in Hospital Affairs (NPHA)	−.538**	.060	.104	.044	.051	.026	.075	.409^**^	1	.464^**^	.380^**^	.468^**^
Staffing and Resource Adequacy (SRA)	−.517^**^	.033	.242^**^	.027	.039	.029	.359^**^	.892^**^	.464^**^	1	.801^**^	.949^**^
Nursing Foundation for Quality Care (NFQC)	−.295^**^	−.051	.165^*^	−.060	−.062	−.068	.280^**^	.749^**^	.380^**^	.801^**^	1	.861^**^
Nursing Practice Environment (NPE)	−.338^**^	.008	.237^**^	.010	.018	.010	.382^**^	.936^**^	.468^**^	.949^**^	.861^**^	1

^∗∗^Correlation is significant at *p* < 0.01 level (2-tailed). ^∗^Correlation is significant at *p* < 0.05 level

“Nurse Manager Ability, Leadership and Support, Nurse-Physician Relation, Nurses’ Participation in Hospital Affairs, Staffing and Resource Adequacy, and Nursing Foundation for Quality Care” are sub-scales of PES-NWI

Table 3 above shows the multiple linear regression model for the predictors of emotional exhaustion of nurses. In model 1, the results showed that the socio-demographic characteristics of nurses accounted for 28.1% variances of emotional exhaustion in nurses, with highest qualification (*B* = -.273, *p* = .000), years in nursing (*B* = .223, *p* = .020) and current rank of nurses (*B* = -.337, *p* = .000) being significant predictors. In the final model, professional practice environment variables were entered explaining an additional 11.5% of the variance in emotional exhaustion. In the final model, all variables were significant predictors of emotional exhaustion explaining 39.6% of the variation (Adjusted *R*^*2*^ = .396, *F* = 14.510, *p* = .000). Nurse manager ability, leadership and support (*B* = -.132, *p* = .023), collegial nurse-physician relations (*B* = .205, *p* = .022), staffing and resource adequacy (*B* = -0.212, *p* = .012), and nurses participation in hospital affair (*B* = -.270, *p* = .001) were important predictors of emotional exhaustion although they differed in their effect.

**Table 3:** Multiple linear regression models for predictors of emotional exhaustion

Model	Unstandardized Coefficients		Standardized Coefficients	T	Sig.
	B	SE	Beta		
**Model 1**					
(Constant)	17.563	9.725		1.806	.072
Age of nurse	.528	.415	.219	1.273	.204
Highest qualification of nurse	−4.063	1.046	−.273	−3.884	**.000**
Years in Nursing	.595	.253	.223	2.350	**.020**
Number of years in hospital	−.779	.636	−.196	−1.225	.222
Current rank of nurse	−2.832	.566	−.337	−5.004	**.000**
**Model 1 Summary: R**^**2**^ **= .281, F =** _**(5, 226)**_ **= 17.622,** ***p*** **= .000**					
**Model 2**					
(Constant)	44.309	10.146		4.367	.000
Age of nurse	.309	.387	.128	.797	.427
Highest qualification of nurse	−2.276	1.019	−.153	− 2.233	**.027**
Years in Nursing	.381	.249	.143	1.531	.127
Number of years in hospital	−.536	.600	−.135	−.894	.372
Current rank of nurse	−1.276	.620	−.152	−2.057	**.041**
Nurse Manager’s Ability, Leadership & Support	−.206	.090	−.132	−2.289	**.023**
Collegial Nurse-Physician Relations	.415	.180	.205	2.302	**.022**
Nursing Foundations for Quality of Care	−.409	.324	−.103	−1.264	.208
Staffing and Resource Adequacy	−.461	.181	−.212	−2.543	**.012**
Nurse Participation in Hospital Affairs	−1.529	.434	−.270	−3.524	**.001**
**Model 2 Summary: R**^**2**^ **= .396, F =** _**(10, 221)**_ **= 14.510, p = .000**					

Outcome: Emotional Exhaustion, 95% confidence level (α = .05)

### Coping strategies used by nurses

The means and standard deviations for coping strategies used by nurses during emotional exhaustion are found in Table 4 below. Nurses reported high usage of the following strategies; ‘I consider several alternatives for handling the problem’ (mean = 3.258), ‘I often try to remember that the problem is not as serious as it seems’ (mean = 3.163), ‘I take steps to take better care of myself and my family for the future’ (mean = 3.150), I make jokes about it or try to make light of it’ (mean = 3.142), ‘I try to see the positive side of the situation’ (mean = 3.137), ‘I make compromises’ (mean = 3.099) ‘I often use exercise, hobbies, or meditation to help me get through a tough time’ (mean = 3.094), and ‘I think about what it might say about bigger lifestyle changes I need to make’ (mean = 3.081).

**Table 4:** Coping strategies used by nurses

Coping Strategy	Min.	Max.	Mean	SD
I spend time trying to understand what happened	1.00	4.00	1.840	.860
I try to see the positive side of the situation.	1.00	4.00	3.137	.731
I try to step back from the problem and think about it from a different point of view.	1.00	4.00	1.879	.874
I consider several alternatives for handling the problem.	1.00	4.00	3.258	.652
I try to see the humor in it.	1.00	4.00	1.745	.827
I think about what it might say about bigger lifestyle changes I need to make.	1.00	4.00	3.081	.748
I often wait it out and see if it doesn’t take care of itself.	1.00	4.00	1.870	.868
I often try to remember that the problem is not as serious as it seems.	1.00	4.00	3.163	.714
I often use exercise, hobbies, or meditation to help me get through a tough time.	1.00	4.00	3.094	.749
I make jokes about it or try to make light of it.	1.00	4.00	3.142	.715
I make compromises.	1.00	4.00	3.099	.734
I take steps to take better care of myself and my family for the future.	1.00	4.00	3.150	.713
I work on making things better for the future by changing my habits, such as diet, exercise, budgeting, or staying in closer touch with people I care about	1.00	4.00	1.754	.824

Source: Field Data (2017)

## Discussion

The study aimed at assessing rate of emotional exhaustion at the practice environment, establishing it determinants and the coping strategies used by nurses to overcome it. It was done among various categories of nursing staff. Most practice environments present occupational threats to the nurse; among factors accounting for such threats are undesirable experience from discomfort and death of patients, conflicts with colleagues and other health professionals, and the absence of support from nurse managers. The nursing profession has therefore been categorised as a risk job for burnout (emotional exhaustion). The study reported higher level of emotional exhaustion (mean = 31.244) with most nurses (*n* = 209, 90.1%) affirming it as indicated in Table 1. Similar studies undertaken at the Sub-Saharan African regions posit that high rates of burnout in a form of emotional exhaustion among nurses and midwives; and are usually attributed to work environments, work conflicts, and lack of social support [31, 47] Other researchers found emotional exhaustion among nurses working in health care facilities in South Africa [48], Ethiopia [49] and Nigeria [50]. It is well established that job stresses results from unsupportive practice environment, and it may affect the nurses’ satisfaction, turnover intentions and the quality of care delivery to patients [51]. The high incidence of emotional exhaustion (90.1%) is consistent with happening in health facilities as most nurses are exposed to stressful challenges in the course of their work such as providing palliative care to end-of-life stages of patients, as well as managing grieving process during death of patient. Again, extremely huge workloads from ones job as a result of the unsupportive work environment can also account for this phenomenon [51, 52]. In other settings, it is reported that high level of abuse from patients and their relatives and some senior members of the health team, dissatisfaction with salaries, limited opportunities for professional improvement and inadequate nurses’ participation in decision making in the hospital account for increased pace of emotional exhaustion among nurses; as these factors devalue the role nurses play in the health care set-up and this can affect them emotionally [9, 53, 54]. Though, much has not been done to curtail emotional exhaustion among nurses in Ghana, effective leadership from nurse managers, monetary compensation from employers [55] and dynamic support from co-workers on daily basis at work place [35] can efficiently decrease this dimension of burnout among nurses in the near future.

The results support the fact that socio-demographic characteristics and facets of professional practice environment of nurses together are predictive of emotional exhaustion (adjusted *R*^*2*^ = 0.396). Regular upgrade of nurses through promotion to higher grades and ensuring nurses’ professional practice environment are vital to developing resilience in managing emotional exhaustion at the work place [56–58].

Additionally, nurses’ perceptions of emotional exhaustion are affected by staffing and resource adequacy in the work place (*B* = -0.212), suggesting that improving staffing and resources in the hospital can reduce emotional exhaustion of nurses by almost third through improving the quantity and quality of nursing human resources and medical equipment used in managing patients. Our findings are consistent with [59, 60] who concluded that poor staffing and resource inadequacy in practice environment are associated with emotional exhaustion of nurses. Institutional policies to enhance adequate staffing and material resources aimed at improving the welfare of nursing staff will have a very important role in reducing emotional exhaustion.

Nursing as a profession expects participation and greater capacity in decision-making in the clients’ care delivery. Therefore, cultivating the spirit of teamwork between health professionals and involving nurses in the management of the health facility would go a long way to eradicate emotional exhaustion and also increase nurses’ self-esteem in health care settings. The current study also found nurse participation in hospital affairs to be significantly predicted with emotional exhaustion (*B* = -0.270). This is consistent with a study which identified that nurses demonstrate more confident when they are involved in collective decision making in health facilities, more so when their professional roles are recognized. Again, work place where nurses and other health professional mutually perform their roles advertently provides foundation for teamwork and enhanced job outcomes [61]. Nurses’ emotional exhaustion can lead to an upsurge of scarcity of nurses, and the consequent poor nursing care delivery to patients. It is, therefore, imperative and critical for health care managers to tackle issues of nurses’ involvement in hospital affairs.

Emotional exhaustion is high in work environment where nurses perceive that their nurse manager are not able to provide adequate leadership and support (*B* = -0.132) for advancement of the nursing profession. Poor perception of the nurse manager ability, leadership and support for nurses corresponds with increase in rates of emotional exhaustion among nurses, as support for most of the stresses nurse encounter is limited. As noted by [62], nursing leadership is valued and respected by subordinates as they expect a great deal of support from their leadership. When nursing leaders fail in their role at influencing nurses’ daily work practice and promoting their welfare through the creation of a positive practice environment; there is always emotional strains on nurses. It is imperative that health facilities find effective approaches to advancing the development and retention of experienced nurse manager to enhance implementation of mechanisms to reduce emotional exhaustion among nurses.

It is an established fact that positive nurse–physician relation at health facilities produces better outcomes for the nurse [63]. A negative correlation was found between nurse-physician relation and emotional exhaustion. It is significant for health care managers, physicians and nurses to ensure efficient communication, mutual support and readiness to compromise having an insightful outcome on teamwork between players in the health care setting. The need to develop ways of building professional respect for team players, promoting productive contact between nurses and physicians and increasing the ability of nursing staff to participate in decision making can go a long way to reduce emotional exhaustion at the work place.

The findings of this study are similar with [64], which suggested the use of a positive coping approach in the form of positive re-appraisal and firmness by attempting to see positive aspects of every challenge and also anxiety reduction approaches by performing recreational activities as remedies for successful stress management. Participating in leisure activities and having an active social life and conversation with relations, friends and peers relieve stress while also elevating the individual’s mood to confront the challenges encountered. As reported, conflict resolution tool of ‘compromise’ was also identified as a means of addressing the emotional exhaustion arising from workplace conflict [65]; and this position is consistent with the study findings. It is significant to note that in order to effectively cope with emotional exhaustion at the workplace, the use of both emotion-focused (e.g. reduction of anxiety and positive re-appraisal) and problem-focused approaches (communicating feeling and support, finding alternative reinforcement) cannot be overlooked. In essence, nurses and nurse managers should identify the best available interventions to mitigate emotional exhaustion in course of their duty.

### Study limitations

The results cannot be generalised to other environments and territories as only five [5] health facilities were used. As with any research study, bias in response can affect the validity of the findings. Our response rate of 92.8%, however, is much higher than that recorded in most nursing surveys, reducing the potential for non-response bias to misrepresent our study findings.

## Conclusions

With far more than 90% of registered nurses experiencing moderate to high emotional exhaustion; it may compromise the quality care of patient. Initiatives such as improved support from nurse manager and good relationship between nurse and physician can positively minimize this phenomenon among nurses. It is also necessary to assess nursing staff’ workload in order to significantly reduce nurses’ emotional exhaustion related to their job-demand and also commit sufficient resources in the training of lower-ranking nurses on how to deal with emotional exhaustion. In an attempt to lessen emotional exhaustion, nurse managers should be accountable for ensuring and promoting reasonable safety in and out of the work environment. Analysing and mitigating risks measures in the sense of organizational structure is essential in building healthy nursing work environments. Furthermore, coping mechanisms when implemented on time can directly influence the interaction between the professional practice environment and the emotional exhaustion of nurses. Thus, when appropriate and effective intervention are employed, emotional exhaustion will be reduce and this will help enrich the quality of care delivered to patients.

## Data Availability

The datasets used and/or analysed during the current study are presented within the manuscript and have also been added as supporting files.

## Abbreviations

CHPS: Community-based Health Planning and Services
GHS: Ghana Health Service
ICUs: Intensive Care Units
LMICs: Low-middle-income countries
IRB: Institutional Review Board
MBI: Maslach Burnout Inventory
PES-NWI: Practice Environment Scale of Nursing Work Index
SDA: Seventh Day Adventist
SSA: Sub-Saharan Africa
WHO: World Health Organization
